# Cryo-EM structure of the *Rhodospirillum rubrum* RC–LH1 complex at 2.5 Å

**DOI:** 10.1042/BCJ20210511

**Published:** 2021-09-07

**Authors:** Pu Qian, Tristan I. Croll, David J.K. Swainsbury, Pablo Castro-Hartmann, Nigel W. Moriarty, Kasim Sader, C. Neil Hunter

**Affiliations:** 1 Materials and Structural Analysis, Thermo Fisher Scientific, Achtseweg Noord 5, 5651 GG Eindhoven, Netherlands; 2 Department of Molecular Biology and Biotechnology, University of Sheffield, Sheffield, U.K.; 3 Cambridge Institute for Medical Research, University of Cambridge, Cambridge CB2 0XY, U.K.; 4 Molecular Biophysics and Integrated Bio-imaging, Lawrence Berkeley National Laboratory, Berkeley, CA 94720, U.S.A

**Keywords:** carotenoids, cryo-electron microscopy, light-harvesting, photosynthesis, quinone, reaction centre

## Abstract

The reaction centre light-harvesting 1 (RC–LH1) complex is the core functional component of bacterial photosynthesis. We determined the cryo-electron microscopy (cryo-EM) structure of the RC–LH1 complex from *Rhodospirillum rubrum* at 2.5 Å resolution, which reveals a unique monomeric bacteriochlorophyll with a phospholipid ligand in the gap between the RC and LH1 complexes. The LH1 complex comprises a circular array of 16 αβ-polypeptide subunits that completely surrounds the RC, with a preferential binding site for a quinone, designated Q_P_, on the inner face of the encircling LH1 complex. Quinols, initially generated at the RC Q_B_ site, are proposed to transiently occupy the Q_P_ site prior to traversing the LH1 barrier and diffusing to the cytochrome *bc*_1_ complex. Thus, the Q_P_ site, which is analogous to other such sites in recent cryo-EM structures of RC–LH1 complexes, likely reflects a general mechanism for exporting quinols from the RC–LH1 complex.

## Introduction

Reaction centre-light harvesting complex 1 (RC–LH1) complexes are the central functional units of photosynthesis in purple phototrophic bacteria. Solar energy absorbed by a circular LH1 assembly migrates to an enclosed membrane-bound RC [1], where a succession of charge separation and protonation events produces a quinol that leaves the RC–LH1 complex, carrying protons and electrons to a cytochrome (cyt) *bc*_1_ complex [2,3]. The LH1 complex is formed from an oligomeric assembly of transmembrane αβ heterodimers, which bind bacteriochlorophyll (BChl) and carotenoid pigments and curve round the central RC complex. In some phototrophic bacteria, the ring of LH1 subunits is incomplete and the ‘missing’ LH1 αβ subunits create a gap through which quinones and quinols can enter and leave the RC–LH1 complex to sustain repeated turnovers of the photosystem. In such complexes one or more transmembrane polypeptides forms part of this interrupted LH1 assembly [4–7]. In other RC–LH1 complexes the RC is fully encircled by LH1 and there is no obvious entry and exit point for quinone traffic [8–10]. Structures of these complexes have revealed small pores in the LH1 antenna that are apparently sufficient to allow the passage of quinones across the LH1 barrier. One such complex, from the phototrophic bacterium *Rhodospirillum* (*Rsp*.) *rubrum*, has been particularly well-studied over many years, with the lack of detailed structural information for this RC–LH1 complex at odds with its important roles in studies of energy transfer [11–13], carotenoid function [14–16] and *in vitro* LH1 assembly [17–19]. Furthermore, more detailed structural data are required to augment earlier NMR work [20] as well as structural information from cryo-electron microscopy (cryo-EM) and atomic force microscopy studies of 2-D crystals [21–24]. A high-resolution structure would also address some intriguing and unsolved aspects of the *Rsp. rubrum* RC–LH1 complex, which include the use of geranylgeraniol (GG) and phytol to esterify the BChl and bacteriopheophytin (BPhe) pigments, respectively [25,26], the shape of the complex that likely imparts curvature on the intracytoplasmic membrane [27,28], and the potentially problematic, continuous LH1 barrier round the *Rsp. rubrum* RC that nevertheless allows rapid quinone traffic between the RC and cyt *bc*_1_ complexes [29].

Here, we used cryo-EM to determine the structure of the RC–LH1 complex from *Rsp. rubrum* at 2.5 Å resolution, which shows the detailed organization of all protein and cofactor components, a preferential site for quinone diffusion across the fully circular LH1 complex, and a unique monomeric BChl, with a phospholipid ligand, in the gap between the RC and LH1 complexes.

## Materials and methods

### Cell culture and protein purification

Wild type cells of *Rsp. rubrum* strain S1 were cultured photosynthetically in M22+ medium under illumination (100 μmol of photons m^−2^ s^−1^) at 30°C in 20 L screw-capped vessels. When the culture reached an absorbance at 680 nm of 1.6 cells were harvested by centrifugation at 3290***g*** for 30 min. The harvested cells were washed using working buffer (20 mM HEPES, pH 7.8). Washed cells were suspended in the working buffer with a few grains of DNase and MgCl_2_ and broken by three passages through a French Press at 18 000 psi. The broken cell suspension was applied to a two-step sucrose gradient (15% and 40% (w/w) in a SW32 ultracentrifuge tube), and spun for 4 h at 100 000***g***. Photosynthetic membranes were collected at the 15%–40% sucrose interface and pelleted. After re-suspension in working buffer, the absorbance of the membrane solution was adjusted to ∼100 at 880 nm. For solubilization of the core complexes, the absorbance at 880 nm of the photosynthetic membrane was adjusted to 60 (1 cm pathlength), and n-Dodecyl-β-d-Maltoside (β-DDM) was added to a final concentration of 3% (w/w). This mixture was then stirred in the dark at 4°C for 30 min. Unsolubilized material was removed by centrifugation for 1 h at 211 000***g***. The clarified supernatant was loaded onto an DEAE-Sepharose ion exchange column pre-equilibrated with running buffer (working buffer solution containing 0.03% β-DDM). The column was washed using 2 column volumes of running buffer followed by stepwise washing to 120 mM NaCl. A 100 ml gradient of 120–300 mM NaCl then was used to elute the complexes from the column. Fractions were monitored using an A880/A820 absorption ratio, and fractions with a ratio >2.0 were pooled, and concentrated for the next purification step on a Superdex 200 gel filtration column. Fractions with an A880/A820 absorbance ratio >2.2 were pooled and used for cryo-EM data collection.

### Cryo-EM data collection

The protein concentration was adjusted to an absorbance of 100 at 880 nm. 3.0 µl protein solution was applied to a glow-discharged holey carbon grid (Quantifoil grid R1.2/1.3, 300 mesh Cu). The grid was plunged into liquid ethane cooled by liquid nitrogen using a FEI Vitrobot 4. Parameters were set as following: blotting time 2.5 s, sample chamber humidity 99%, sample chamber temperature 4°C. The frozen grid was stored in liquid nitrogen before use. Data were recorded at the Cambridge Pharmaceutical Cryo-EM Consortium on a ThermoFisher Scientific Titan Krios G3i Cryo-Transmission Electron Microscope (Cryo-TEM) equipped with a Falcon 4 direct electron detector. The microscope was operated at 300 kV accelerating voltage, at a nominal magnification of 120 000×, corresponding to a pixel size of 0.65 Å at the specimen level. The detector was operated in counting mode. A total dose of 45 electrons per Å^2^ was divided between 42 frames within 12.21 s exposure time, resulting in an electron dose of 1.07 e^−^/Å^2^/frame. In total, 9 024 movies were collected with defocus values varied from 0.8 to 2.2 µm. A typical cryo-EM image, averaged from motion corrected movie frames, is shown in Supplementary Figure S1A.

### Data processing

Image processing was performed within RELION 3.1. Beam-induced movement of dose fractionated images were corrected using RELION's built-in motioncorr2 [30] on 5 × 5 patches. CTF parameters were determined using CTFFIND4 [31]. In total, 1 519 688 particles were picked based on the particle coordinates calculated from cisTEM [32] with a box size of 380 × 380, corresponding to a 24.7 nm square. These particles were subjected to reference-free two-dimensional classification. 1 128 646 (74%) particles from good 2D classes were selected for 3D classification. The resulting good 2D classes were subjected to an initial 3D model calculation using EMAN2 [33] for maximum-likelihood-based 3D classification. The best 3D class out of four, containing 519 005 particles (34%), was selected for high resolution 3D reconstruction and refinement, resulting in a 3.2 Å resolution 3D map. After CTF refinement, including anisotropic magnification, beam-tilt, trefoil, fourth order aberration, per particle defocus and per-image astigmatism estimation, Bayesian polishing was performed with the default parameters provided by RELION, improving the resolution of the 3D map to 2.7 Å. The selected particles for the 3D refinement were re-extracted using a 512 × 512 box size for a final CTF refinement and Bayesian polishing, producing a 2.5 Å resolution map.

### Modeling and refinement

Initially, the crystal structure of the reaction centre from *Rhodobacter* (*Rba.*) *sphaeroides* (PDB 3I4D) was fitted to the cryo-EM map as a rigid body using the *fit in map* function of Chimera [34]. The polypeptides of the RC-H, RC-M and RC-L subunits were mutated according to those from *Rsp. rubrum* using COOT [35]. They were then manually adjusted and real space refined for both polypeptides and cofactors. For LH1, a single subunit of the LH1 complex, αβBChl_2_Car, was built based on structural similarity compared with the LH1 of *Thermochromatium* (*Tch.*) *tepidum* [36]. It was docked into the density map so that the two BChl *a* molecules in the model fitted comfortably into their density in the map. Amino acid sequences then were replaced with those from *Rps. rubrum*, and real space refined in COOT. This subunit was copied to the other 15 αβ subunits, forming a closed LH1 ring. Real space refinement was performed on individual αβ subunits. All-*trans* spirilloxanthin and ubiquinone-10 molecules (Q_B_ and Q_P_) were also fitted into the density map independently using COOT.

Analysis of pigment composition shows that the major carotenoid in the core complex is spirilloxanthin [37]; BChl *a* is esterified by a GG tail [38], whereas BPhe is esterified by phytol. *Rsp. rubrum* contains two types of quinone, ubiquinone-10 and rhodoquinone-10 [39]. All-*trans* spirilloxanthin and 15-*cis* spirilloxanthin fitted well into the LH1 and RC, respectively. BChl *a*_GG_ was used for the LH1 BChl *a* pair, RC special pair and RC accessory BChl *a*. Based on the 2.5 Å resolution map, RC Q_A_ and Q_B_ and Q_P_ were assigned to UQ-10, whereas Q_F_ was assigned to RQ-10. In addition, two tetramyristoyl cardiolipin and two phosphatidylglycerol lipids were modeled into the map. Restraints for novel ligands were generated using eLBOW [40] with the ideal values for bonds and angles obtained from Mogul [41,42]. All of the LH1 subunits and ligands underwent real-space refinement using ISOLDE [43]. The final model was subjected to global refinement and minimization using PHENIX [44]. The final refinement statistics are summarized in Supplementary Table S1. The quality of fit for the structural model within the electron density map was validated using EMRinger [45].

## Results and discussion

### Overall structure of the RC–LH1 complex

**Figure 1.**
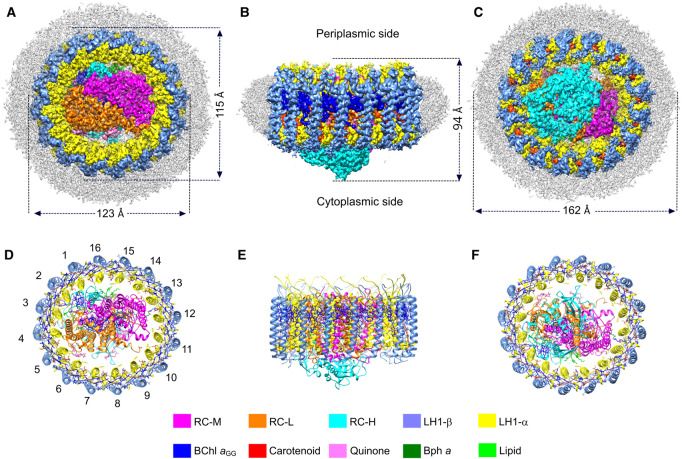
Cryo-EM structure of the RC–LH1 core complex from *Rsp. rubrum*. (**A**–**C**) Views of the RC–LH1 density map, colored as in the key at the bottom of the figure. Detergent and other disordered molecules are in grey. (**A**) View of the slightly elliptical LH1 ring from the periplasmic side of the membrane, showing the diameters of the long and short axes. (**B**) View in the plane of the membrane showing the height of the complex. (**C**) Perpendicular view from the cytoplasmic side. (**D**–**F**) Ribbon models corresponding to (**A**–**C**), made using Chimera [58]; the LH1 subunits are numbered in (**D**).

In this study, we report the structure of *Rsp. rubrum* RC–LH1 complex at 2.5 Å, determined by cryo-EM. In total, 9,024 cryo-EM movies were recorded, from which 1 519 688 particles were picked for further data processing yielding a final resolution of 2.5 Å (Figure 1, Supplementary Figures S1, S2, Supplementary Table S1). The density map (Figure 1A–C) shows the overall shape of the complex, which is slightly elliptical in projection with short and long axes of 115 and 123 Å (Figure 1A), and 94 Å in height (Figure 1B). As Figure 1B also shows, the shape of the complex in the plane of the membrane is cylindrical, yet the chromatophore membranes of *Rsp. rubrum*, in which RC–LH1 complexes are the dominant complex [46], are curved with a ∼50–100 nm diameter [27,28]. Only a few degrees of conical angle are required to account for this level of curvature, and we conclude that in the native membrane a ring of lipid molecules binds to the outer face of the LH1-β polypeptide on the cytoplasmic side of the complex. Inspection of the density map shows disordered detergent molecules in the position that lipids are suggested to occupy, and lipids bound tightly in these positions round the LH1 ring would confer a slight conical shape on the complex. There are precedents for bound, and structurally resolved, rings of lipids and detergents in RC–LH1 complexes from *Rhodopseudomonas (Rps.) palustris* (RC–LH1_14_-W; Protein Data Bank (PDB) 6Z5S, and RC–LH1_16_, 6Z5R) [6], *Rba. veldkampii* (7DDQ) [7], *Tch. tepidum* (5Y5S) [9] and *Thiorhodovibrio* (*Trv.*) strain 970 (7C9R) [10]. Ribbon models of the complex are shown in Figure 1D–F. Although the RC is completely surrounded by the LH1 complex only one H-bond connects the RC and the LH1 ring, between LH1-α1 Ser34 and RC-H Trp9 (3.1 Å); we suggest that this point of contact is a potential site for initiating the assembly of a curved array of LH1 αβ subunits that culminates in a fully encircled RC, so it is assigned as αβ(1). Each of the 16 pairs of transmembrane α and β polypeptides, numbered in Figure 1D, binds two BChl *a*_GG_ molecules creating a ring of 32 closely spaced and paired BChl *a* pigments. In this respect, the *Rsp. rubrum* LH1 antenna is very similar to the 16-subunit ring of *Tch. tepidum* [9] and to the RC–LH1_16_ complex of *Rps. palustris* [6].

### Structure of the LH1 complex

**Figure 2.**
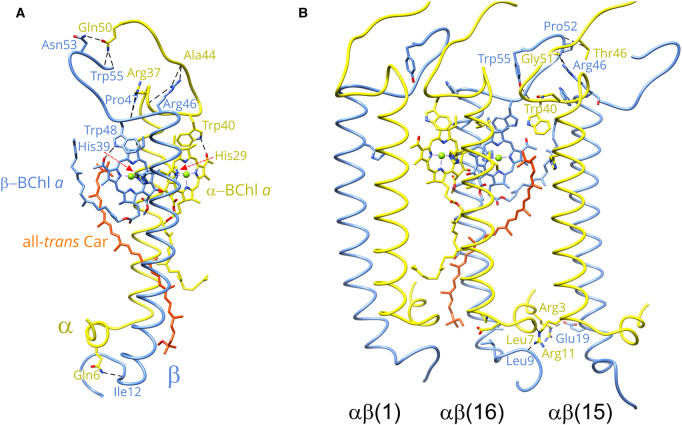
Protein–protein and protein–pigment interactions in the *Rsp. rubrum* RC–LH1 core complex. (**A**) A single LH1 αβ subunit, containing one α-polypeptide (yellow), one β-polypeptide (cornflower blue), two BChl *a*_GG_ molecules colored according to their cognate polypeptide, and one all-*trans* spirilloxanthin (red-orange). All residues involved in H-bonds are labelled. (**B**) Inter-subunit interactions between three adjoining LH1 αβ subunits. Pigments are only shown in the middle subunit for clarity. Only residues involved in inter-subunit H-bonds are labelled.

The LH1 αβ subunits are the building blocks of the LH1 ring; Figure 2A shows the structure of a single αβ subunit, which is stabilized by a series of protein–protein, BChl–protein, BChl–BChl, carotenoid–BChl and carotenoid–protein contacts. LH1α consists of a short N-terminal helix of nine residues that lies parallel to the surface of the membrane on the cytoplasmic side, followed by a transmembrane domain and a 14-residue C-terminal region that contains a loop structure. The LH1 β-polypeptide has the same topology as LH1α. Hydrogen bonds between C- and N-terminal residues link the α and β polypeptides and contribute to the formation of LH1 αβ subunits: α-Gln50—β-Asn53, 2.9 Å; α-Gln50—β-Trp55, 2.9 Å; α-Ala44—β-Arg46, 2.9 Å; α-Arg37—β-Arg46, 2.8 Å; α-Arg37—β-Pro47, 2.8 Å and α-Gln6—β-Ile12, 2.8 Å. However, the binding of BChl and carotenoid pigments, and the ensuing pigment-pigment interactions, are the major driving forces that create the LH1αβ(BChl)_2_ units, which behave in a modular fashion that can form either open or closed rings [4–10], or, in some RC-minus mutants, ellipses, and spirals of variable size [47]. LH1α His29 and LH1β His39 supply the ligands (2.6 and 2.5 Å, respectively) for binding a pair of opposing, excitonically coupled BChls. These pigments are modeled with GG ‘tails’ (BChl *a*_GG_) in the well-resolved density map (Supplementary Figure S2). As with other light-harvesting LH1 complexes, hydrogen bonds from C-terminal residues (LH1α Trp40 and LH1β Trp48) to BChl C3 acetyl groups (2.6 and 2.5 Å, respectively) tune the absorption properties of the bound BChls [48,49]. A combination of excitonic coupling within and between BChl dimers, together with hydrogen bonding to LH1-α and LH1-β aromatic side chains, redshifts the absorption from ∼770 nm for monomeric BChl in solvent to 880 nm in the RC–LH1 complex. The progressive red-shifting of absorption, as more LH1αβ(BChl)_2_ units associate, has been examined extensively using *in vitro* reconstitution approaches [17–19]. Some of these studies employed a carotenoid-free mixture of polypeptides and BChls, but the native complex necessarily binds carotenoids, both for harvesting light in the 450–550 nm region of the spectrum and for photoprotection. Many studies of excited state dynamics have focused on the *Rsp. rubrum* LH1 complex, for example [11–14], but the location and conformation of the carotenoid, spirilloxanthin, have remained unknown. Here, the structure of the RC–LH1 complex shows that the spirilloxanthins in the LH1 ring are in the all-*trans* configuration, making contacts with the N-terminal helical region (α3-RIWQLF) of the n − 1 α-polypeptide and the transmembrane region (α26-LLIHFILL) of the n + 1 α-polypeptide. The central region of the spirilloxanthin is in close contact (4.2 Å) with the tail of the α-BChl *a*_GG_ (Figure 2B). The extent of these contacts reveals a third, stabilizing, role for the carotenoid, analogous to the same role for these pigments in LH2 complexes [50,51]. Thus, carotenoids stabilize individual LH1αβ subunits, and interactions between spirilloxanthins and n + 1, n, n − 1 polypeptides interlock LH1 αβ subunits and promote the formation of a curved, oligomeric assembly. A subset of this interlocking ring structure is shown in Figure 2B, in which hydrogen-bonds help to stabilize associations between adjacent LH1αβ subunits. Focusing on subunits 15 and 16, at the periplasmic (C-terminal) side of the complex these bonds are between β(15)-Gly51 and β(16)-Arg46, 3.0 Å; β(15)-Pro52 and α(16)-Thr46, 3.3 Å; β(15)-Tyr55 and α(16)-Trp40, 2.6 Å. On the N-terminal side there are hydrogen bonds between α(15)-Glu19 and β(16)-Arg3, 2.8 Å; α(15)-Leu7 and α(16)-Arg11, 2.8 Å; β(15)-Leu 9 and α(16)-Arg 11, 2.8 Å.

### The reaction centre, and the space between this complex and LH1

**Figure 3.**
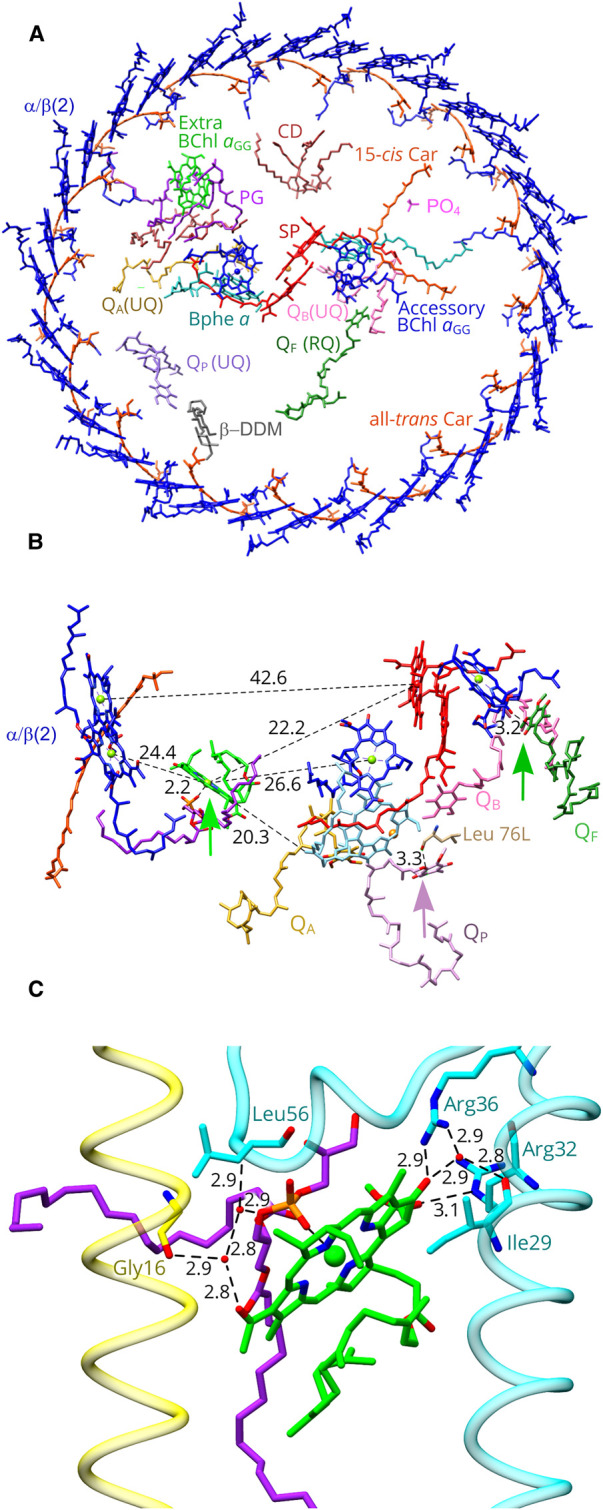
Arrangement of pigments and cofactors in the *Rsp. rubrum* RC–LH1 core complex. (**A**) Perpendicular view from the periplasmic side of the membrane. Water molecules were omitted for clarity. PG, Phosphatidyl glycerol; CD, cardiolipin; SP, the reaction centre special pair of BChl *a*_GG_ molecules. (**B**) Location and pigment environment of the extra BChl *a*_GG_ (bright green; colours as in panel (**A**)). The central Mg ion of this BChl *a*_GG_ is coordinated by the nearby PG lipid molecule, indicated by a bright green arrow. A lone pair π interaction between Q_P_ and RC-L Leu 76 is indicated with a mauve arrow, and a mid-green arrow points to a lone pair π interaction between non-protein bound rhodoquinone RQ and the accessory BChl *a*_GG_ on the B-branch of the RC. (**C**) Detailed view of the bonding environment for the extra BChl *a*_GG_ (green). Three water molecules are shown as small red spheres, with hydrogen bonds shown as dashed lines with the distances in Ångstroms indicated. LH1 α is in yellow, The RC-H subunit is in cyan, and the PG is in magenta.

The RC consists of H, M and L subunits (Figure 1, Supplementary Figure S2), and the internal arrangement of BChl, bacteriopheophytin (BPhe), carotenoid (Car), quinone and Fe cofactors is shown in Figure 3A. Supplementary Figure S2 shows the quality of fits of atomic models for all the cofactors and protein components to their respective densities within the 2.5 Å resolution map. The unusual presence of both GG and phytol ‘tails’ of the various RC pigments in *Rsp. rubrum* has been known for many years [25]. BChl *a* esterified with a GG tail (Supplementary Figure S3), BChl *a*_GG_, was used in our structural model for the RC special pair and accessory BChl pigments; the BPhe pigments are esterified with phytol. Surprisingly, we identified an extra BChl *a*_GG_, localized between the second LH1αβ subunit and the RC special pair (Figure 3A). This pigment is not bound to a protein, and instead it is coordinated by an oxygen within the headgroup of a neighboring phosphatidylglycerol molecule. This type of coordination has not been observed before in RC–LH1 complexes, but in the RC from the green sulfur bacterium *Chlorobaculum tepidum* [52] it has been proposed that the headgroup of a phosphatidylglycerol molecule coordinates a water molecule forming the axial ligand of the A0 Chl *a* [53]. One of the acyl chains of the RC-LH1 lipid is close to the GG tail of a BChl bound to the α-polypeptide of LH1αβ(2) and to a spirilloxanthin molecule (Supplementary Figure S4). Presumably the interactions between LH1, a lipid, and the extra BChl are sufficient to position this pigment consistently, so it becomes a defined feature in the density map.

The function of this BChl aGG pigment, unique in currently determined RC–LH1 structures, is unknown. Figure 3 displays the distances to neighboring pigments, showing that the extra BChl *a*_GG_ is located approximately midway between the BChl *a*_GG_ pair attached to LH1αβ(2) and the RC special pair of BChl *a*_GG_ pigments, a distance of 42.6 Å, with its macrocycle tilted ∼60° from the special pair. The distance from the special pair is 22.2 Å, and 26.6 Å separates the extra BChl *a*_GG_ from the accessory BChl *a*_GG_ on the active (A branch) of the bifurcated network of RC cofactors. In the context of the inverse sixth power distance dependence for Förster energy transfer [54], halving the gap between the LH1 donor and RC acceptor pigments could exert a significant effect on LH1 to RC transfer. However, the absorption maximum of this pigment is also a factor to consider. Figure 3C shows the environment of this extra BChl *a*_GG_ which, in addition to its ligand with the nearby phosphatidyl glycerol (magenta), forms a series of hydrogen bonds with the RC H-subunit and with the second LH1α subunit. The C13^2^ ester of this BChl *a*_GG_ hydrogen-bonds to RCH-Arg32 NE, and the C13^1^ keto bonds to RCH-Arg36. In addition, three water molecules (small red spheres in Figure 3C) form a hydrogen bond network between the C3-acetyl of BChl *a*_GG_, the backbone oxygen of LH1α–Gly16, the lipid and the backbone N of RCH–Leu56. Finally, there is also a water bridging to the RCH–Ile29 OH. As a monomeric pigment, the absorption maximum of this extra BChl *a*_GG_ is likely to be ∼800 nm by analogy with, for example, LH2 complexes [55,56]. A protein engineering study was conducted on the β-Arg30 residue near to the monomeric B800 BChl in the LH2 complex of *Rba. sphaeroides*. Raman spectroscopy of β-Arg30 mutants showed that the loss of a hydrogen bond to the C3-acetyl group, and it was concluded that the absorption red-shift attributable solely to the H-bond is ∼10 nm [55]. Thus the absorption maximum of a monomeric BChl, ∼780 nm in solvent, would likely absorb no further to the red than 800 nm and this is proposed to apply to the BChl *a*_GG_ in the *Rsp. rubrum* RC–LH1 complex. If this is the case, energy transfer from 880 nm LH1 BChls to this new BChl would be ‘uphill’; onward excitation transfer to the RC special pair absorbing at 865 nm would be correspondingly ‘downhill’, though, and energetically level to the accessory RC BChl absorbing near 800 nm. Overall, the extra BChl *a*_GG_ could act as a conduit for energy transfer to the RC, but early picosecond transient absorption experiments showed no difference between *Rsp. rubrum* and *Rba. sphaeroides* RC–LH1 complexes, with rates of energy transfer in the 30–40 ps range [12].

Four quinone molecules were identified: Q_A_, Q_B_ and Q_p_ are assigned as ubiquinone-10, based on their well-resolved densities (Supplementary Figure S2). Both the RC Q_A_ and Q_B_ sites are occupied, and there is a clear density for another quinone that sits between the RC and the inner face of the LH1 complex. Despite the disorder presumed to exist in the space between the RC and LH1 complexes, we could assign a rhodoquinone-10 at a position designated Q_F_ (‘free’ quinone) based on the match between its chemical structure and the density shape (Figure 3A, Supplementary Figures S2, S3). ‘Free’ quinones, with no surrounding protein to provide a defined binding pocket, were also found in *Rps. palustris*, *Tch. tepidum* and *Rba*. *veldkampii* RC–LH1 complexes [6,7,9]. There is a lone pair π interaction between the head of Q_F_ and the C13^1^ acetyl group of the accessory BChl *a*_GG_ on the B-branch.

**Figure 4.**
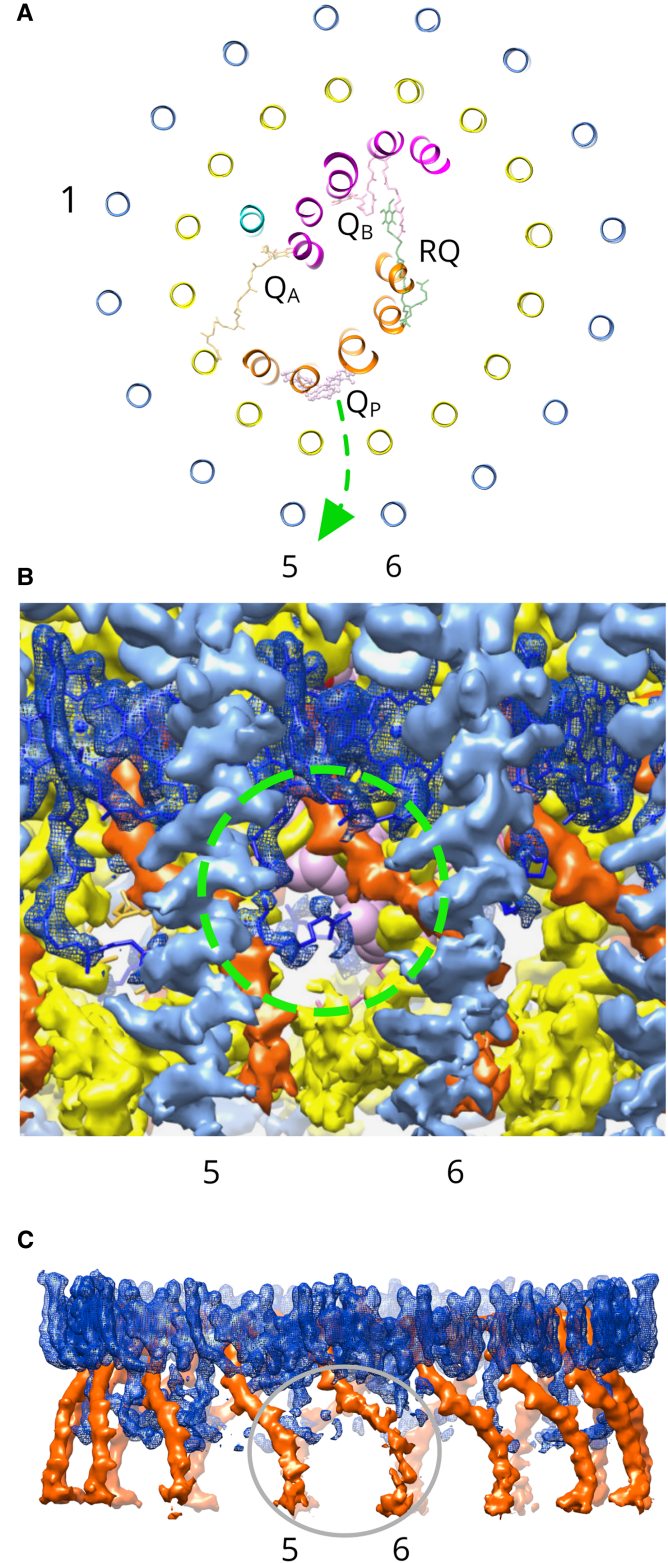
Location of a quinone/quinol channel adjacent to the Q_P_ site in the LH1 ring. (**A**) The RC–LH1 complex viewed from the periplasmic side, with α-helices represented as ribbons and appearing as circles in the case of LH1 polypeptides. Only quinone cofactors are shown. Subunits are colored as in Figure 1. LH1-αβ subunits 1, 5 and 6 are labeled. A green arrow indicates the proposed path taken by a quinol though the pore. (**B**) View of the LH1 complex in the plane of the membrane, from outside the complex. The LH1 α-polypeptide is in yellow, the β-polypeptide in cornflower blue, BChl *a*_GG_ molecules are blue, and the all-*trans* spirilloxanthin is in red-orange. The green dashed circle shows the pore, with the weak density of this particular GG tail allowing a view of the Q_P_ quinone (pink) visible in the background. (**C**) Densities of pigments adjacent to the LH1 pore. The grey ellipse delineates the weaker densities for the GG tail and the carotenoid at these positions in the LH1 ring.

In the *Rsp. rubrum* structure, there is one spirilloxanthin molecule per LH1αβ(BChl)_2_ unit, and this stoichiometry has been proposed to create a series of pores round the LH1 ring that could allow passage of quinone across the LH1 ring by means of ‘breathing motions’ [29,55]. This view of quinone diffusion does not assign any particular location or set of LH1 subunits for traversing the LH1 ring. However, the *Rsp. rubrum* RC–LH1 structure adds to the lengthening list of complexes [5–10] that have a conserved Q_P_ quinone (Figure 4A). The head group of the Q_P_ quinone in the *Rsp. rubrum* LH1 makes lone pair π interactions with the backbone oxygen of RC-L Leu 76, and the isoprenyl tail makes contacts with LH1α4 and 5. As with other such Q_P_ sites, we suggest that transient docking into this binding pocket prepares the quinone for passage through an adjacent pore between LH1αβ(BChl)_2_ units. This does not rule out migration paths across other parts of the LH1 ring, although these routes may be less probable. In particular, we note the structurally resolved β-DDM detergent molecule (Figure 3A, grey), which fits well into the density near to Q_P_ (Supplementary Figure S5). This detergent molecule could have displaced a lipid, or a quinone, or it could sit in a pore in the LH1 ring between subunits 6 and 7, indicating another possible point of exit for quinol produced at the RC Q_B_ site.

The Q_P_ pore is circled in green in Figure 4B, and the Q_P_ quinone molecule can be seen by virtue of the weaker density for the GG tail of the BChl, relative to other pigments in the ring (Figure 4C). Similarly, the density for the cytoplasmic end of the spirilloxanthin in LH1αβ (6) is also weaker than for neighboring carotenoids. Counterparts of the Q_P_ quinone have been found in several RC–LH1 structures [5–10], and in each case Q_P_ associates with the inner face of the LH1 complex adjacent to a pore that would allow quinone movement across the LH1 ring. In the case of the *Blastochloris viridis* RC–LH1 [8], Q_P_ is adjacent to a region of relatively weak density for the BChl phytol and the associated carotenoid, which was proposed to reflect conformational flexibility leading to local disorder where quinones diffuse across the complex. We propose that transient occupation of the Q_P_ site increases the probability that the quinol will pass through the adjacent pore in the RC–LH1 complex of *Rsp. rubrum*.

## Data Availability

The cryo-EM density map has been deposited in the World Wide Protein Data Bank (wwPDB) under accession code EMD-13110 and the coordinates have been deposited in the Protein Data Bank (PDB) under accession number 7OY8.

## Open Access Statement

Open access for this article was enabled by the participation of University of Sheffield in an all-inclusive *Read & Publish* pilot with Portland Press and the Biochemical Society under a transformative agreement with JISC.

## Abbreviations

β-DDM: n-Dodecyl-β-d-Maltoside
BChl *a*_GG_: BChl *a* esterified with a GG tail
BChl: Bacteriochlorophyll *a*
BPhe: bacteriopheophytin
cryo-EM: cryo-electron microscopy
GG: geranylgeraniol
*Rba.*: *Rhodobacter*
RC–LH1: reaction centre light-harvesting 1 complex
*Rps.*: *Rhodopseudomonas*
*Rsp*.: *Rhodospirillum*
*Tch.*: *Thermochromatium*
*Trv.*: *Thiorhodovibrio*
