# Supplementary material for: Cryo-EM structure of the Rhodospirillum rubrum RC–LH1 complex at 2.5 Å
Source: Biochem J. 2021 Sep 7;478(17):3253–63. doi: 10.1042/BCJ20210511 (PMC8454704; doi:10.1042/BCJ20210511)
Supplement: Supplementary Figures S1-S5 and Table S1 [file BCJ-478-3253-s1.pdf]

Supplementary material

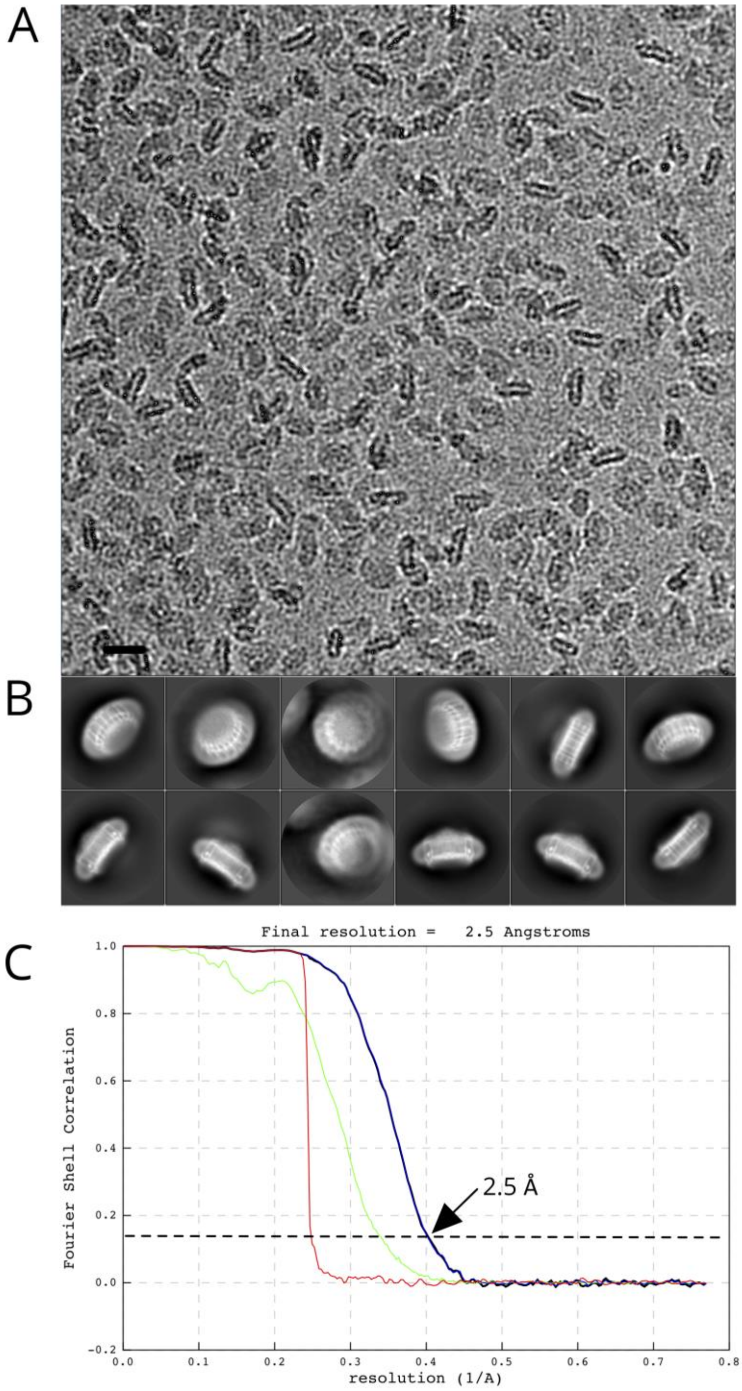

Figure S1. Cryo-EM image of the RC-LH1 complex from *Rsp. rubrum* and resolution calculation of 3D map of the complex. (A) A selected cryo-EM micrograph after motion correction. The pixel size of the image is 4k x 4k, corresponding to 266 x 266 nm at the specimen level. A 20 nm scale bar is indicated. (B) Selected 2D classes, box size 25 nm, showing different views of the complex. (C) Fourier shell correlations (red, corrected, phase randomized; green, unmasked; blue, masked). The dashed line shows 0.143 FSC, with an arrow pointing to the global resolution of the map at 2.5 Å.

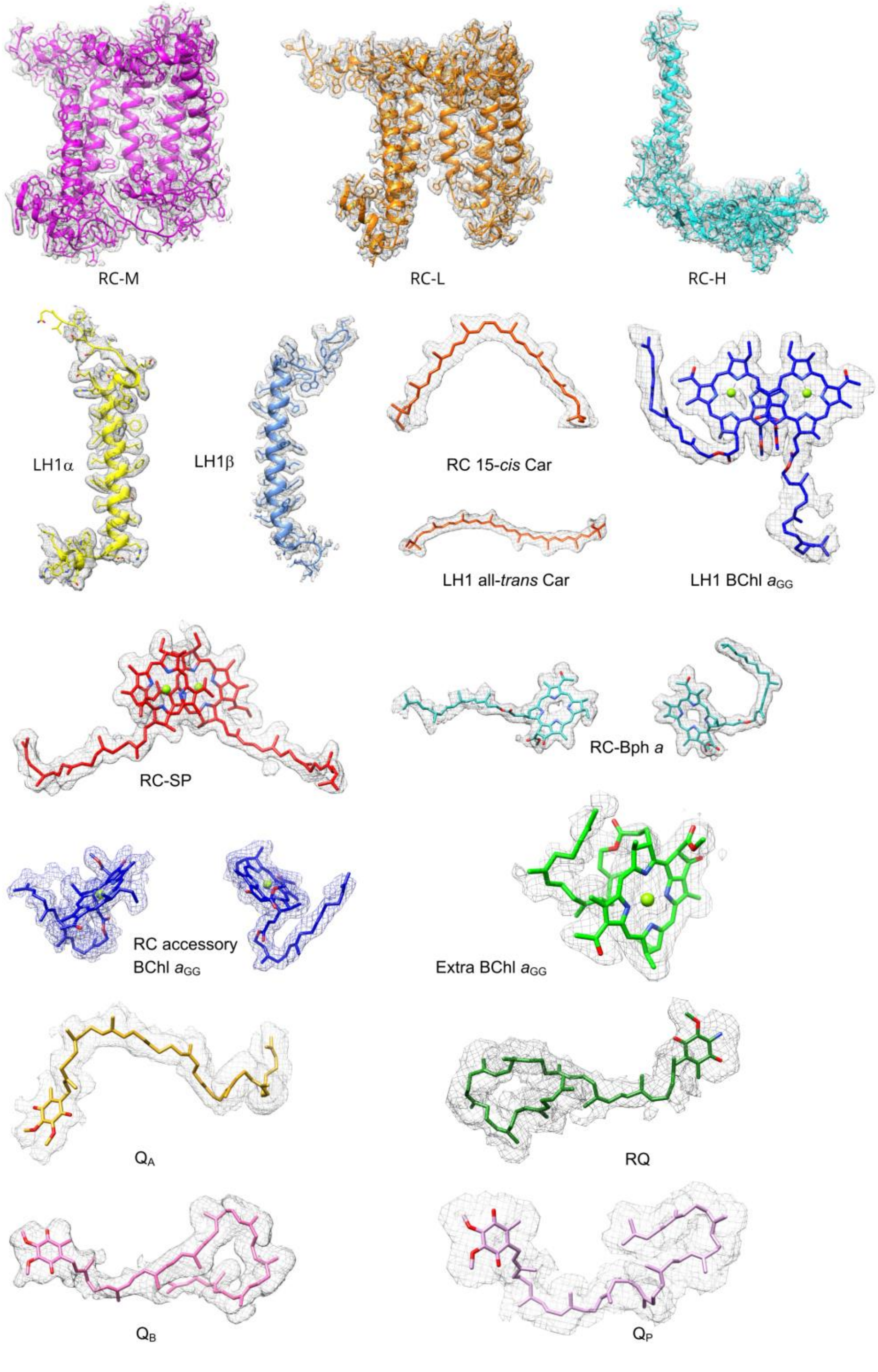

Figure S2. Cryo-EM densities and structural models of polypeptides and pigments in the *Rsp. rubrum* RC-LH1 complex. Atomic models of components of the complexes are fitted into their respective density maps, taken from the final refined model. Color codes are the same as in Figure 1.

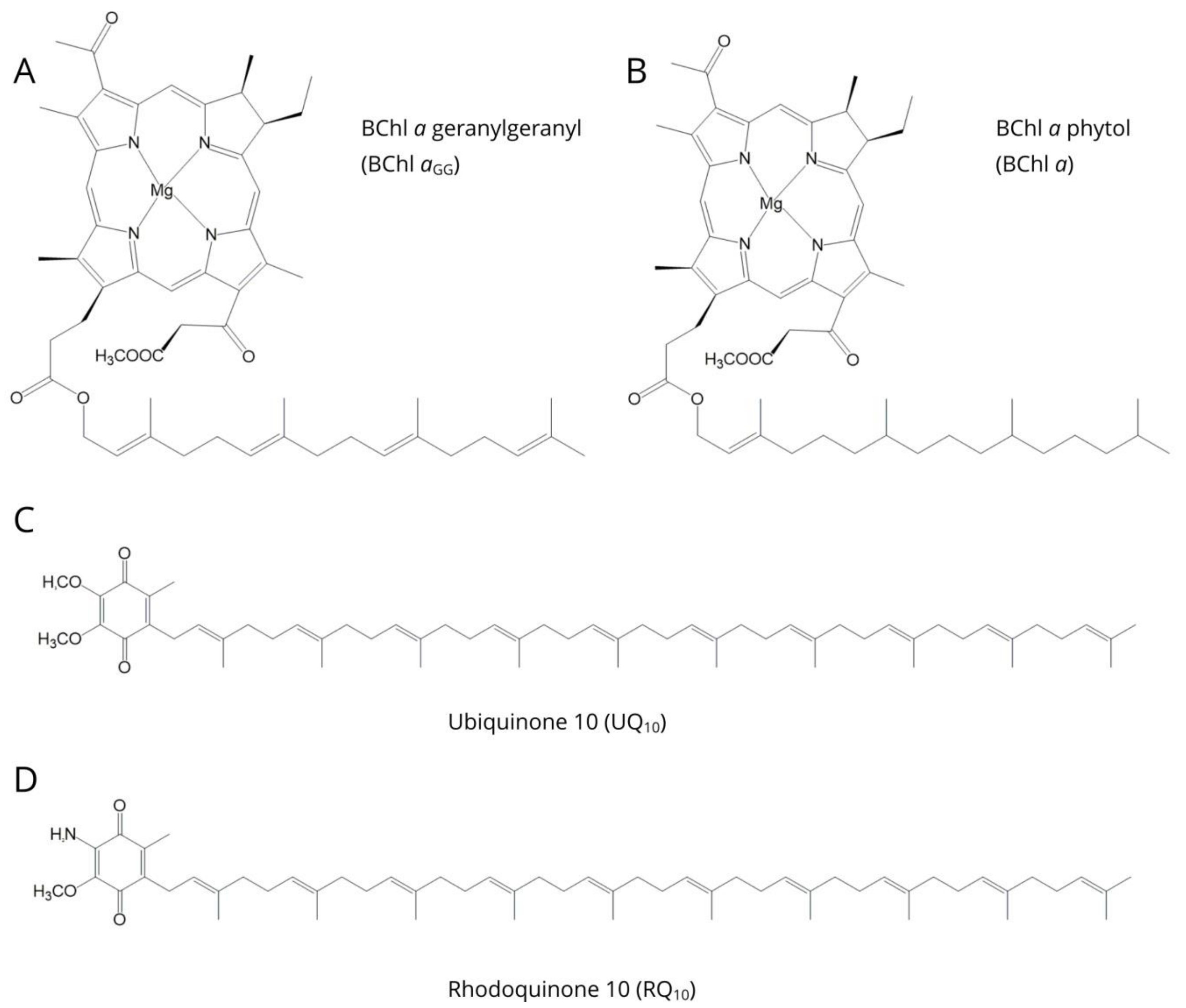

Figure S3. Chemical structures of different bacteriochlorophyll *a* and quinone molecules in the RC-LH1 complex of *Rsp. rubrum*. (A) Bacteriochlorophyll *a* esterified with a geranylgeraniol tail. (B) Bacteriochlorophyll *a* esterified with a phytol tail. (C) Ubiquinone-10. (D) Rhodoquinone-10.

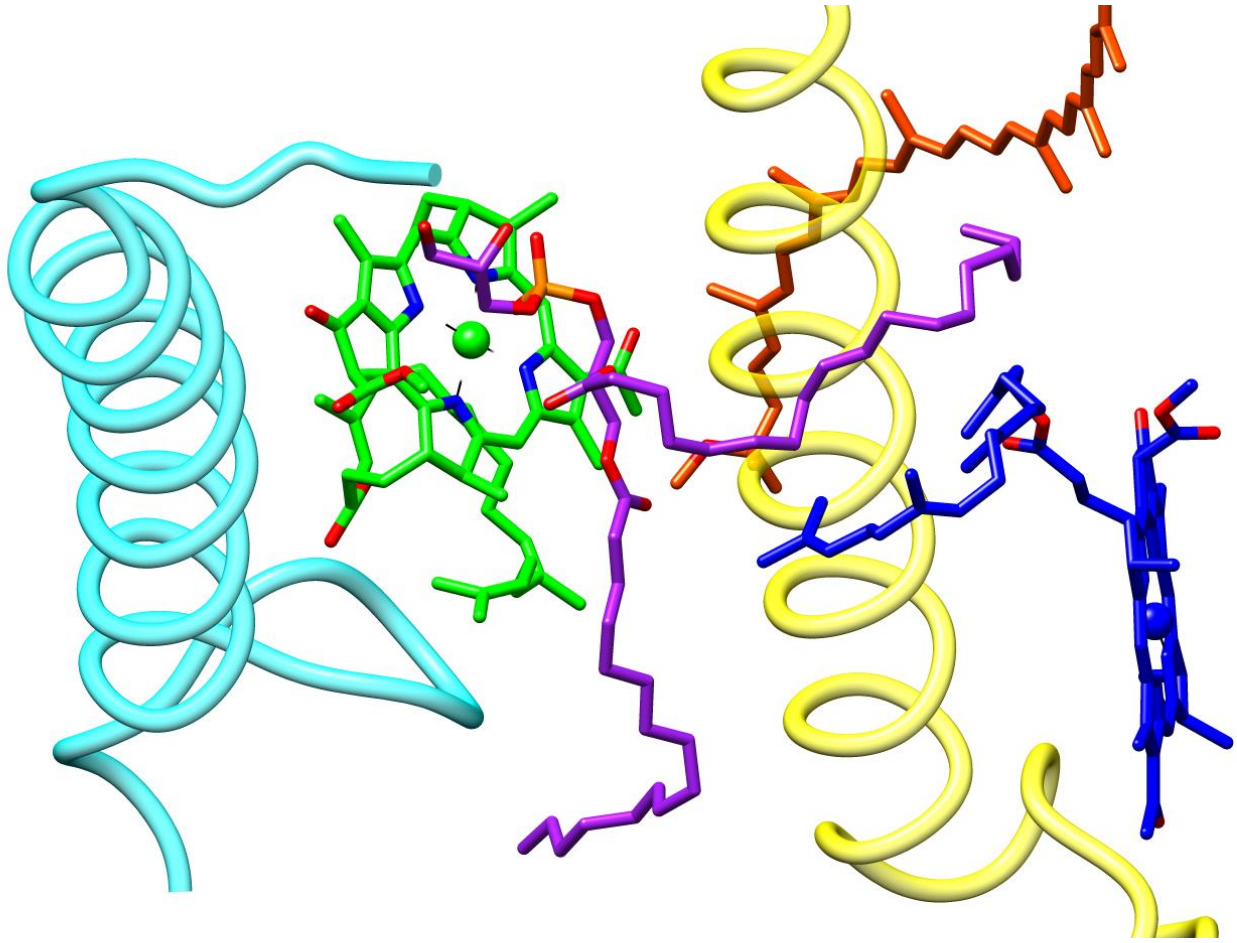

Figure S4. Positioning of the phosphatidyl glycerol molecule in relation to the extra BChl a_{GG}, the nearest carotenoid and the GG tail of an LH1 BChl. Colours: phosphatidyl glycerol (magenta), the extra BChl a_{GG} (green), LH1 BChl a_{GG} (blue) LH1 α (yellow), RC-H subunit (cyan).

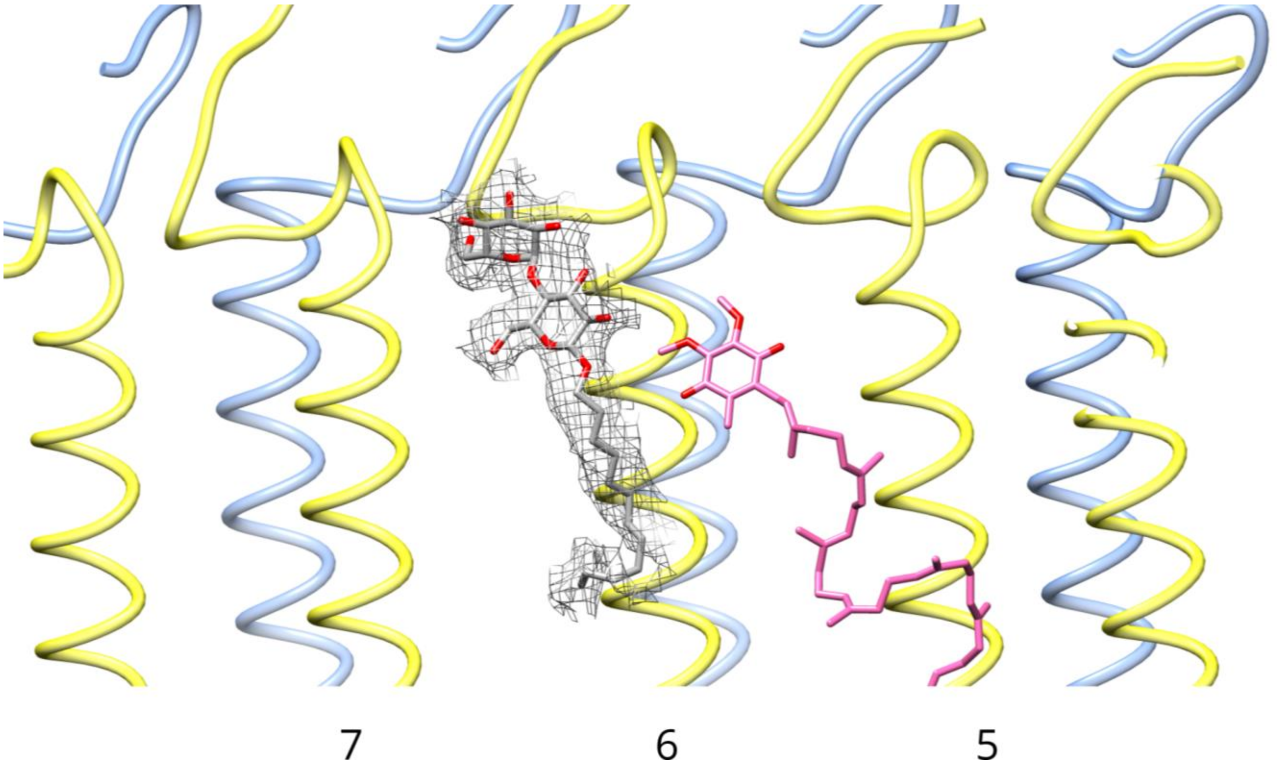

Figure S5. Density for the structurally resolved β-DDM detergent molecule (grey), near to Q_P (pink). These molecules are viewed from the inside of the LH1, looking out. The β-DDM detergent molecule lies between LH1 subunits 6 and 7, and Q_P is between subunits 5 and 6. Colours: β-DDM detergent (grey), Q_P (pink), LH1α (yellow), LH1β (blue).

Table S1: Cryo-EM data acquisition, model refinement and validation statistics.

**Protein source**	Photosynthetic bacterium
**Data collection and processing**	
Microscope	ThermoFisher Titan Krios G3i
Voltage (kV)	300
Camera	Falcon 4
Energy filter	No
Energy filter slit width	N/A
Magnification	120,000
Defocus range (µm)	-0.8 to -2.2
Mean defocus (µm)	-1.8
Pixel size (Å)	0.65
Electron flux (e^-/ $Å^2$/s)	3.71
Electron fluence (e^-/ $Å^2$)	44.94
Exposure time (sec/frame)	0.29
Electron fluence per frame (e/ $Å^2$/frame)	1.07
Number of frames per movie	42
Number of movies used	9024
Initial no. particle images	1,519,688
Final no. particle images	519,005
Estimated accuracy of translations (Å) (RELION)	0.32
Estimated accuracy of rotations(^{o}) (RELION)	0.98
Symmetry imposed	C1
Local resolution range	2.2 to 2.8
Resolution of unmasked reconstruction (Å, FSC=0.143)	2.85
Resolution of masked reconstruction (Å, FSC=0.143)	2.5
Specimen temperature	~80K
Particle box size	$(512\ px)^2$
**Refinement and validation**	
Refinement package	COOT, ISOLDE, PHENIX
Initial model	PDB 1LGH, PDB 3I4D
Model resolution (Å, FSC=0.5)	2.5
Map sharpening B factor ($Å^2$)	-72.38
**Model composition**	
Non-hydrogen atoms	22902
Protein residues	2293
Molecular weight (kD)	297.7
Protein B factor ($Å^2$)	27.74
**RMS deviations**	
Bond length (Å)	0.003
Bond angle (^{0})	0.752
**Validation**	
MolProbity score	0.79
Clashscore	0.65
Rotamer outliers (%)	0.21
EMRinger score	6.29
Cb deviations (%)	0.00
CaBLAM outliers (%)	0.4
**Ramachandran plot**	
Favoured (%)	97.64
Allowed (%)	2.36
Disallowed (%)	0.00
Ramachandran Z-score	0.49
**PDB ID**	7OY8
**EMDB ID**	EMD-13110
